# A Newly Identified lncBCAS1-4_1 Associated With Vitamin D Signaling and EMT in Ovarian Cancer Cells

**DOI:** 10.3389/fonc.2021.691500

**Published:** 2021-08-05

**Authors:** Yaqi Xue, Ping Wang, Fei Jiang, Jing Yu, Hongmei Ding, Zengli Zhang, Hailong Pei, Bingyan Li

**Affiliations:** ^1^Deparment of Nutrition and Food Hygiene, Medical College of Soochow University, Suzhou, China; ^2^Department of Clinical Nutrition, The First Affiliated Hospital of Soochow University, Suzhou, China; ^3^Department of Obstetrics and Gynecology, The First Affiliated Hospital of Soochow University, Suzhou, China; ^4^State Key Laboratory of Radiation Medicine and Protection, School of Radiation Medicine and Protection, Collaborative Innovation Centre of Radiological Medicine of Jiangsu Higher Education Institutions, Soochow University, Suzhou, China

**Keywords:** vitamin D, long noncoding RNAs, transcriptome, lncRNA-mRNA network, EMT, lncBCAS1-4_1

## Abstract

Long noncoding RNAs (lncRNAs) were identified rapidly due to their important role in many biological processes and human diseases including cancer. 1α,25-dihydroxyvitamin D3 [1α,25(OH)_2_D_3_] and its analogues are widely applied as preventative and therapeutic anticancer agents. However, the expression profile of lncRNAs regulated by 1α,25(OH)_2_D_3_ in ovarian cancer remains to be clarified. In the present study, we found 606 lncRNAs and 102 mRNAs that showed differential expression (DE) based on microarray data. Gene Ontology (GO) and Kyoto Encyclopedia of Genes and Genomes (KEGG) pathway analysis indicated that the DE genes were mainly enriched in TGF-β, MAPK, Ras, PI3K-Akt, and Hippo signaling pathways, as well as the vitamin D-related pathway. We further assessed the potential lncRNAs that linked vitamin D signaling with EMT, and lncBCAS1-4_1 was identified in the first time. Moreover, we found that the most upregulated lncBCAS1-4_1 showed 75% same transcripts with CYP24A1 (metabolic enzyme of 1α,25(OH)_2_D_3_). Finally, the lncBCAS1-4_1 gain-of-function cell model was established, which demonstrated that the knockdown of lncBCAS1-4_1 inhibited the proliferation and migration of ovarian cancer cells. Furthermore, lncBCAS1-4_1 could resist the antitumor effect of 1α,25(OH)_2_D_3_, which was associated with upregulated ZEB1. These data provide new evidences that lncRNAs served as a target for the antitumor effect of 1α,25(OH)_2_D_3_.

## Introduction

Ovarian cancer is the leading cause of death caused by gynecologic malignancies (1). Despite the significant medical advances during the past decades, the 5-year survival rate of ovarian cancer is lower than 50% (2). Long noncoding RNAs (lncRNAs), with transcripts more than 200 nucleotides in length, were suggested to play fundamental roles in the development of tumor (3), due to the fact that lncRNAs may exhibit tumor suppressive or promoting functions through the regulation of transcription, translation, protein modification, and the formation of RNA–protein or protein–protein complexes (4). Therefore, lncRNAs are possible candidates of cancer biomarkers and/or therapeutic targets (5). Recently, 13 published papers investigated the expression of lncRNA in normal ovaries, ovarian cysts, and benign and malignant ovarian cancer (6–18), suggesting the important role of lncRNAs in ovarian cancer development and chemotherapeutic survival outcomes of patients. Thus, it is important to explore the potential of lncRNAs as a therapeutic target of ovarian cancer.

Accumulating evidences supported the role of vitamin D in reducing cancer risk and improving prognosis. The active metabolite of vitamin D, 1α,25 dihydroxyvitamin D3 [1α,25(OH)_2_D_3_], has been demonstrated to have an anticancer effect by inhibiting proliferation, inflammation, invasion, metastasis, and angiogenesis. In addition, 1α,25(OH)_2_D_3_ could promote apoptosis and differentiation (19, 20) through mitogen-activated protein kinase (MAPK), NF-κB, and PI3K−Akt-dependent signaling pathways (21–23). These findings indicate that 1α,25(OH)_2_D_3_ may play an important role in the control of cancer by mediating protein-coding genes. In addition, one and two reports studied vitamin D receptor (VDR)-regulated lncRNAs profiling in skin cancer and breast cancer, respectively (24–26). However, it is not clear which lncRNAs are regulated by 1α,25(OH)_2_D_3_ in ovarian cancer.

In the present study, the lncRNA and mRNA networks were constructed using microarray data, which were used to explore the profile of lncRNA in 1α,25(OH)_2_D_3_-treated human ovarian cancer SKOV3 cells comprehensively. Moreover, the potential lncRNAs that linked vitamin D signaling with EMT were analyzed, and lncBCAS1-4_1 was identified. Besides, the effect of lncBCAS1-4_1 on the proliferation and migration in 1α,25(OH)_2_D_3_-treated ovarian cancer cells was investigated.

## Materials and Methods

### Microarray Expression Profiling

SKOV3 cells were treated with 1α,25(OH)_2_D_3_ (100 nmol/L) or vehicle (the same concentration of ethanol) for 72 h. Total RNA was extracted with a TRIzol reagent (Thermo Fisher Scientific, Scotts Valley, CA, USA) and quantified using NanoDrop™ ND-2000 (Thermo Fisher Scientific). After RNA integrity was assessed using an Agilent Bioanalyzer 2100 (Agilent Technologies, CA, USA), sample labeling, microarray hybridization, and washing were performed based on the manufacturer’s standard protocols (OE Biotech Company, Shanghai, Design ID: 076500). Briefly, total RNA was transcribed to double-stranded cDNA and then synthesized into cRNA, which was labeled with Cyanine-3-CTP. The labeled cRNAs were hybridized onto the microarray. After washing, the arrays were scanned by the Agilent Scanner G2505C (Agilent Technologies).

### Differentially Expressed Gene Analysis

Limma (Version 3.8) package in R software was used to identify the differently expressed mRNAs (DE-mRNAs) and -lncRNAs (DE-lncRNAs) with a threshold of |log2 (fold change [FC])| > 2.0 and a false discovery rate [FDR (adjusted *p*-value)] < 0.05. The heatmap and volcano were constructed by the gplots package in R software.

### Functional Enrichment Analysis

To reveal the functions of DE genes, the Enricher database was used to conduct Gene Ontology (GO) annotation and Kyoto Encyclopedia of Genes and Genomes (KEGG) pathway enrichment analyses (27). The GO terms comprised of the following three divisions: biological process (BP), cellular component (CC), and molecular function (MF). A significance level of *p* < 0.05 was set as the cutoff criterion, and the plots were constructed by the gplots package in R software.

A PPI network of DE mRNAs was constructed using STRING 11.0 (http://string-db.org), with a combined score > 0.9 as the cutoff value. Significant modules in the PPI network were identified using MCODE 1.5.1 (a Cytoscape software plug-in).

### Construction of the lncRNA-mRNA Co-Expression Modules

The lncRNAs and mRNAs co-expression modules were further selected using Pearson correlation analysis. The lncRNA–mRNA pairs with a correlation coefficient > 0.9 and *p* < 0.05 were used for bidirectional clustering.

### Enrichment of EMT Signal Pathway in Ovarian Cancer Cells Treated With 1α,25(OH)_2_D_3_

Here, 200 EMT-related genes were downloaded from the Molecular Signature Database v7.1 (MSigDB) (http://www.broad.mit.edu/gsea/msigdb/). To identify the EMT pathway involved in 1α,25(OH)_2_D_3_-treated ovarian cancer cells, risk signature was used in Gene Set Enrichment Analysis (GSEA), and *p* < 0.05 and FDR > 2 were considered as statistically significant.

### Quantitative Real-Time PCR

Reverse transcription reactions consisted of 1 μg RNA and 2 μL of 5xPrimerScript RT Master Mix (TaKaRa, Japan) with a total volume of 10 μL. The primer sequences of RNA are shown in **Table 1**. Reactions were performed in a C100 PCR System (Bio-Rad, Hercules, CA, USA) for 15 min at 37°C. GAPDH was used as the internal control. The qPCR was performed using the SYBR Green (Roche, Basel, Switzerland) dye detection method on the ABI 7500 PCR instrument (Applied Biosystem, Foster City, CA, USA) under default conditions: 95°C for 10 s, 40 cycles of 95°C for 5 s, and 55°C for 30 s. The relative gene expression levels were analyzed by the 2^-ΔΔCt^ method, where ΔCt = Ct(target) – Ct(GADPH).

**Table 1:** Primer sequences for PCR.

Primers	5’-3’
lncBCAS1-4_1	F GGTAAGGGTGGGCTGGATTT
	R TGGAGTTGATGCTAGTGAATCCC
lncZNF599-3_6	F CCTCCGCTGACTTCAACCAA
	R CACTTCAAAACTGCAAAAAGGGC
lncMBOAT1-4_2	F GGAGAGTCACAGCTGGTCAA
	R GATGAGATGGCTCTGGGATGG
lncRWDD4-5_1	F GGACTTTGCTGTCGTGGGAC
	R CCGTAAAAGCACTGGCCGTAA
lncKRT7-2_2	F TGAAGTAGGAAGGAACGCAGC
	R CAGCTCACTTGTTCAGGGGT
CYP24A1	F CGCATCTTCCATTTGGCGTT
	R GCCTGGATGTCGTATTTGCG
E-cadherin	F CCCACCACGTACAAGGGTC
	R CTGGGGTATTGGGGGCATC
N-cadherin	F TGGCTCCACTGCTGGGTCCT
	R GCCAAAGCCTCCAGCAAGCA
Vimentin	F TACAGGAAGCTGCTGGAAGG
	R ACCAGAGGGAGTGAATCCAG
Twist1	F GCAAGATTCAGACCCTCAAGC
	R TCCATCCTCCAGACCGACAA
ZEB1	F TTCAAACCCATAGTGGTTGCT
	R TGGGGAGATACCAAACCAACTG
Slug	F CGAACCCACACATTGCCTTG
	R GTGAGGGCAAGAGAAAGGCT
GAPDH	F AGCCACATCGCTCAGACAC
	R GCCCAATACGACCAAATCC

### Construction of lncBCAS1-4_1 Loss/Gain Cell Model

Overexpression adenoviruses (OE) as well as control adenoviruses (empty vector, EV) of lncBCAS1-4_1 were purchased from GeneChem Corporation (Shanghai, China). The knockdown lncBCAS1-4_1 was produced by siRNA interference. Scramble control and silncBCAS1-4_1 were purchased from RiboBio Co., Ltd (Guangzhou, China) and transfected using riboFECT™ CP (Guangzhou, China) according to the manufacturer’s instructions. All oligonucleotide sequences are listed in **Table 2**.

**Table 2:** Target sequences of siRNA.

siRNA	Sense 5′- 3′
si-h-Lnc-BCAS1-4_001	GCTAAATGAACGGTCTGTA
si-h-Lnc-BCAS1-4_002	GCTGCGGTGGAAACGGTAA
si-h-Lnc-BCAS1-4_003	GGATTCACTAGCATCAACT

### Cell Proliferation Assay

Cell colony formation and the CCK-8 counting were used to assay cell proliferation, respectively. Briefly, 3×10^4^ OVCAR8 or 2.5×10^4^ SKOV3 cells were seeded onto 60-mm culture plates and transfected by adenoviruses or siRNAs for 72 h. After the cells were treated with 1α,25(OH)_2_D_3_ (100 nmol/L) or ethanol for 48 h, they were fixed with 75% alcohol and stained with 0.3% methyl violet for 20 min at room temperature. Then the colonies were dissolved by glacial acetic acid, and the absorbance value (AU) was detected at 585 nm with a microplate reader (Filter Max F5, Molecular Devices, CA, USA). The cell proliferation ratio was calculated as (AU_treatment group_ – AU_blank group_)/(AU_control group_ – AU_blank group_). All experiments were performed in triplicate.

According to the manufacturer’s instructions, approximately, 3×10^3^ OVCAR8 or 2.5×10^3^ SKOV3 cells per well were plated in triplicate into 96-well plates and treated with 1α,25(OH)_2_D_3_ for 48 h. The control group was treated with ethanol. At each of the desired time points, 10 μL of the CCK-8 solution was added and incubated for 1 h at 37°C, followed by measurement of absorbance at 450 and 630 nm with a microplate reader for quantifying the relative cell density. Cell viability was calculated as: (AU _450-treatment group_ – AU_630-treatment group_)/(AU_450-control group_ – AU_630-blank group_). All experiments were performed in triplicate.

### Cell Migration Assay

The cell migration was assessed using a wound healing assay. Cells were plated into a 6-well plate with FBS-free media for 12 h. Afterwards, cells cultured in the bottom of the well were scratched using a pipette tip to create a wound area. After 24 and 48 h, wounds (three images each well) were imaged under a microscope (40, CKX41F, Olympus, Tokyo, Japan) to detect the width of the gaps. Wound healing assay data are displayed as the migration index (%), which is calculated by the formula [(initial width) - (final width)]/(initial width). Values were normalized by the control group. Data points in the figure represent three independent experiments.

### Statistical Analysis

All microarray statistical data were analyzed in the R environment (R version: 3.6.3). Wilcoxon/Mann–Whitney test was used to analyze continuous variables, and Fisher’s exact test or chi-square test was used to analyze the categorical data. Experimental data were performed using GraphPad Prism 8 (GraphPad Software Inc., La Jolla, CA, USA). Quantitative data were presented as the mean ± standard deviation (SD). Statistical data were analyzed using an unpaired Student’s t-test. For all statistical analyses, a *p*-value less than 0.05 was regarded as statistically significant.

## Results

### Identification of Differentially Expressed lncRNA and mRNAs in 1α,25(OH)_2_D_3_ treated SKOV3 Cells

**Figure 1:**
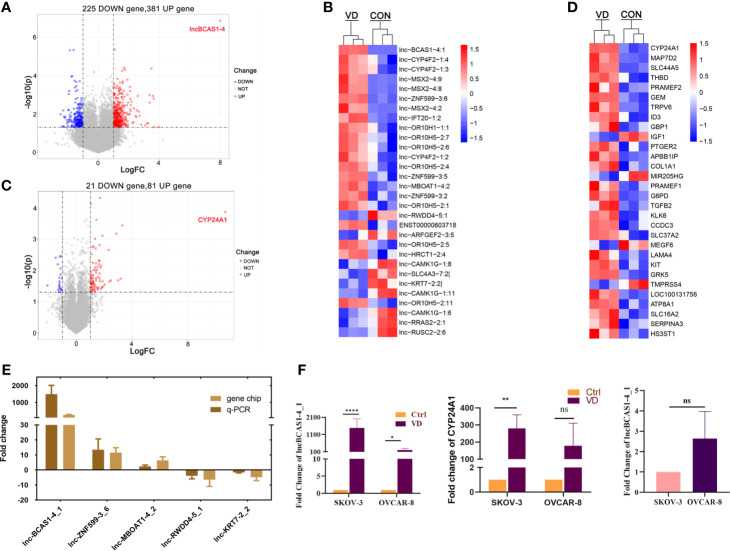
Distributions of differentially expressed genes in ovarian cancer cells treated with 1α,25((OH)_2_D_3_. The ascending normalized expression level in the heatmaps is colored from blue to red. Red means gene upregulation, blue indicates downregulation, and white means normal expression. Furthermore, each column represents a sample, and each row represents a differentially expressed gene. **(A)** The volcanoes of 606 DE lncRNAs; **(B)** the heatmaps of DE-lncRNAs; **(C)** the volcanoes of 102 DE-mRNAs; **(D)** the heatmaps of DE-mRNAs; **(E)** the validation of DE-lncRNAs by quantitative RT-PCR; and **(F)** the expressions of most-regulated lncBCAS-1_4-1 and CYP24A1 in SKOV3 and OVCAR8 treated by 1α,25(OH)_2_D_3_. *p < 0.05, **p < 0.01, ****p < 0.0001, NS, not significant.

To identify the differentially expressed (DE) mRNAs and lncRNAs in human ovarian cancer cells, SKOV3 cells were treated with 1α,25(OH)_2_D_3_ or vehicle to obtain lncRNA and mRNA expression profiles by microarray analysis (GSE 173633). Compared with control cells, 606 lncRNAs were dysregulated (fold change > 2.0, *p* < 0.05) in 1α,25(OH)_2_D_3_-treated SKOV3 cells, in which 381 lncRNAs were upregulated and 225 lncRNAs were downregulated. lnc-BCAS1-4_1 was the most upregulated lncRNA, with a 257.77-fold change (**Figure 1A, B**). In addition, among 102 dysregulated mRNAs, there were 81 upregulated mRNAs and 21 downregulated mRNAs. Interestingly, the most upregulated mRNA transcript was CYP24A1, the metabolic enzyme of 1α,25(OH)_2_D_3_, and the fold change was 1653.22 (**Figures 1C, D**).

To further validate the findings of the microarray analysis results, five dysregulated lncRNAs were confirmed using quantitative RT-PCR. lnc-BCAS1-4_1 and lnc-RWDD4-5_1 were selected as target lncRNAs with the most upregulated/downregulated expression. Lnc-ZNF599-3_6 was selected for its potential function of trans-regulating, and the other two (lnc-MBOAT1-4_2 and lnc-KRT7-2_2) were randomly selected. Consistently, their expressions from quantitative RT-PCR results were similar with those of the microarray analysis (**Figure 1E**). Similarly, the transcriptional levels of lncRNABCAS1-4_1 and CYP24A1 were indeed dramatically increased after 1α,25(OH)_2_D_3_ treatment (**Figure 1F**). We searched with BLAST and found that lncBCAS1-4_1 showed 75% same transcripts with CYP24A1 (**Supplementary Data Figure S1**). Interestingly, the fold change is more remarkable in SKOV3 cells, which might be linked with the lower background of lncBCAS1-4_1 in SKOV3 cells (**Figure 1F**), indicating that the expression of lncBCAS1-4_1 could have influence on the efficacy of 1α,25(OH)_2_D_3_.

### Vitamin D-Regulated lncRNA–mRNA Network in Ovarian Cancer Cells

**Figure 2:**
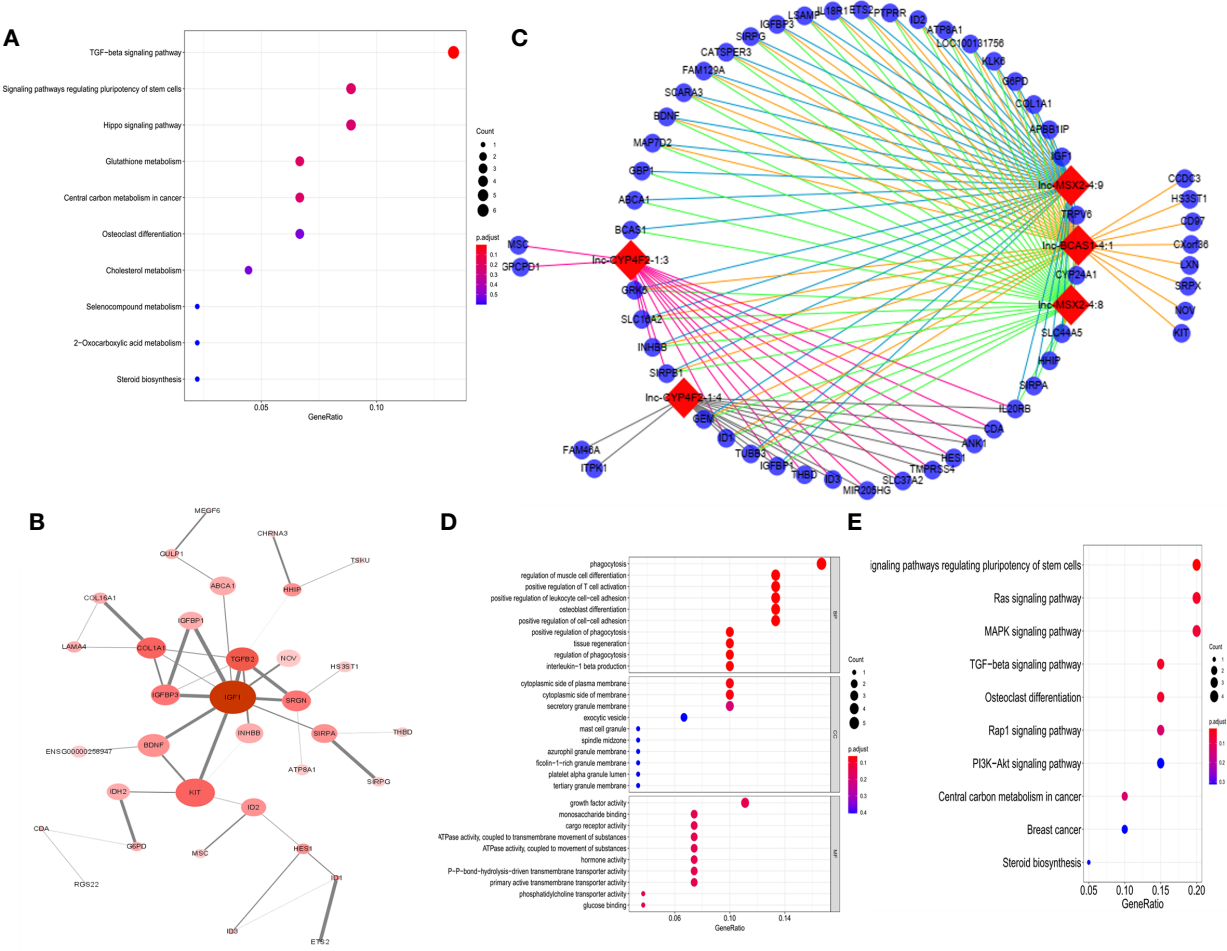
Function analysis of DE-RNAs. **(A)** The KEGG pathways from total DE-mRNAs, mean enrichment of genes in different pathways. The y-axis represents the pathways, and the x-axis represents enriched gene numbers; the color means adjusted p-value; the most important pathways. **(B)** The most significant hub genes identified by protein–protein interaction (PPI). **(C)** Vitamin D-regulated lncRNA–mRNA network including top 5 lncRNAs and 140 mapped mRNAs. The red nodes represent top 5 differentially expression lncRNAs, and the blue nodes represent mapped mRNA. **(D)** GO analysis contains the biological process (BP), cellular component (CC), and molecular function (MF). The x-axis represents the gene ratio, and the y-axis represents GO terms. The size of the circle indicates the gene count, and the color means adjusted p-value. **(E)** The KEGG pathways from top 5 lncRNA regulated-DE mRNAs. The y-axis represents the pathways, and the x-axis represents enriched gene numbers; the color means adjust p-value.

To explore the potential responsible mechanism of cancer cells for 1α,25(OH)_2_D_3_, KEGG pathway analysis was performed on the DE genes. The results indicated that DE mRNAs were mainly enriched in TGF-β, regulating pluripotency of stem cells, and Hippo signaling pathways (**Figure 2A**). The hub genes with a degree connectivity in PPI network were enriched in insulin-like growth factor 1 (IGF1), which is known to induce cell proliferation (28), TGF-β2 (29), insulin-like growth factor-binding protein 3 (IGFBP3) (28), and COL1A1 (30), which are closely associated with the vitamin D endocrine system (**Figure 2B**). Then, we identified a top 5 lncRNAs–mRNAs networks including 5 lncRNAs and 140 mapped mRNAs (**Figure 2C** and **Supplementary Data Table S1**). GO enrichment analysis and subpathway analysis showed that “phagocytosis”, “cytoplastic side of plasma membrane”, and “growth factor activity” were significantly related to this module (**Figure 2D**). KEGG analysis for 140 mRNA from top 5 lncRNA–mRNAs networks revealed that cancer-related pathways were enriched in this network, e.g., Ras, MAPK, TGF-β, Rap1, and PI3K-Akt signaling pathway (**Figure 2E**).

### Construction of the lncBCAS-1_4-1 as a Core of EMT Signal Pathway in 1α,25(OH)_2_D_3_ Treated Ovarian Cancer Cells

**Figure 3:**
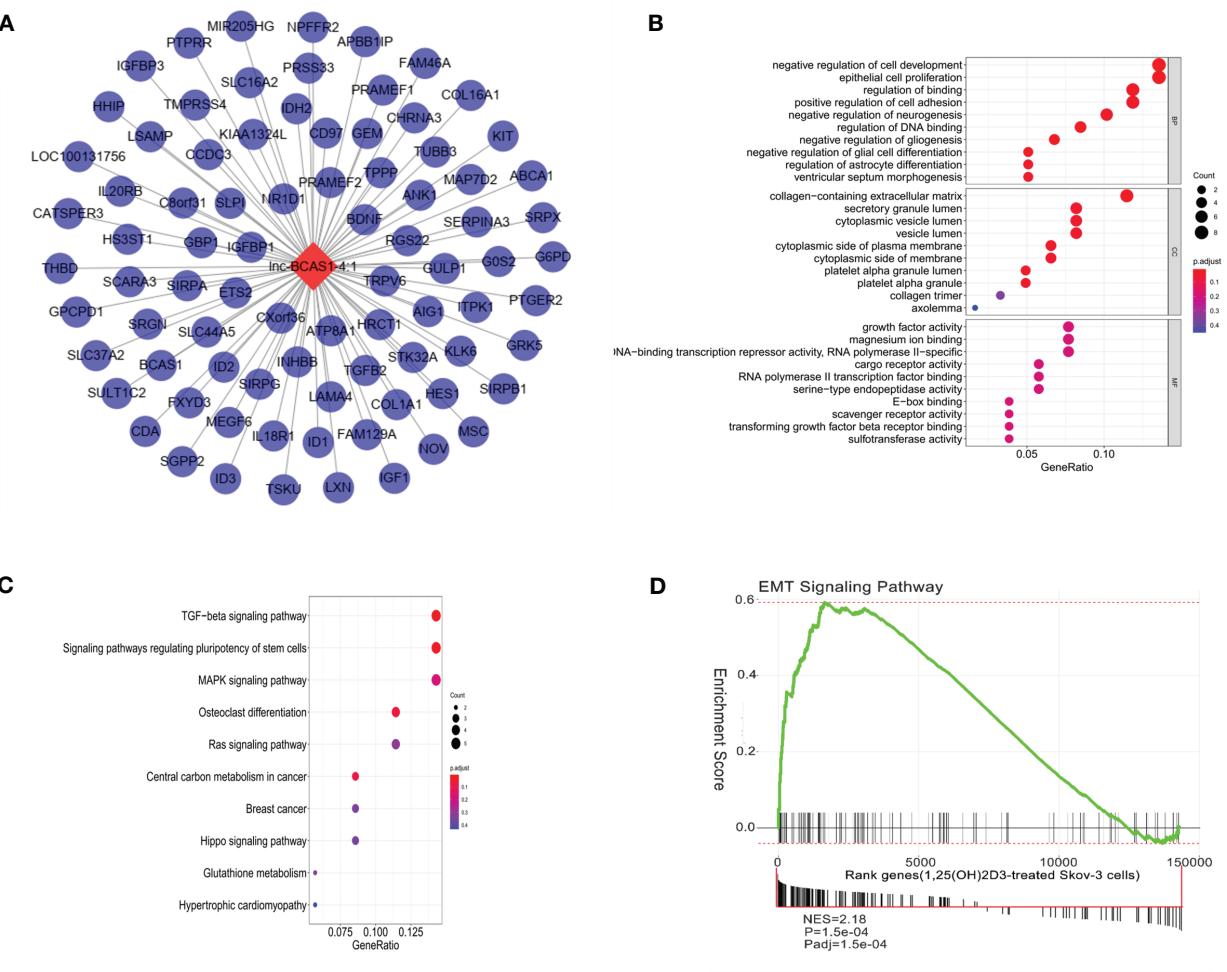
Function analysis of lncBCAS1-4_1. **(A)** lncBCAS1-4_1-mRNA networks. The red nodes represent top differentiated expression lncBCAS1-4_1, and the blue nodes represent mapped mRNA. **(B)** GO analysis contains the biological process (BP), cellular component (CC), and molecular function (MF). The x-axis represents the gene ratio, and the y-axis represents GO terms. The size of the circle indicates the gene count, and the color means adjusted p-value. **(C)** The KEGG pathways from lncBCAS1-4_1 regulated DE-mRNAs. The y-axis represents the pathway, and the x-axis represents enriched gene numbers; the color means adjust p-value. **(D)** Gene set enrichment analysis for EMT-related lncRNA signature in 1α,25(OH)_2_D_3_-treated SKOV3 cells.

Next, we selected the most dysregulated lncRNA, lncBCAS1-4_1, to construct the lncRNA–mRNA network, and 83 mapped mRNAs were involved to explore the function of this module (**Figure 3A** and **Supplementary Data Table S2**). GO analysis showed that “epithelial cell proliferation”, “collagen-containing extracellular matrix”, and “growth factor activity” were highly enriched in this network (**Figure 3B**). KEGG analysis also revealed that these genes mainly enriched in TGF-β, regulating pluripotency stem cells, MAPK, Ras, and Hippo signaling pathways (**Figure 3C**). Because the TGF-β signaling pathway repeatedly occurred, the EMT-related genes were applied to identify the significant pathway associated with 1α,25(OH)_2_D_3_; as shown in **Figure 3D**. The EMT pathway was significantly activated in this network (**Figure 3D**).

### The Role lncBCAS-1_4-1 on Proliferation and Migration of Ovarian Cancer Cells

**Figure 4:**
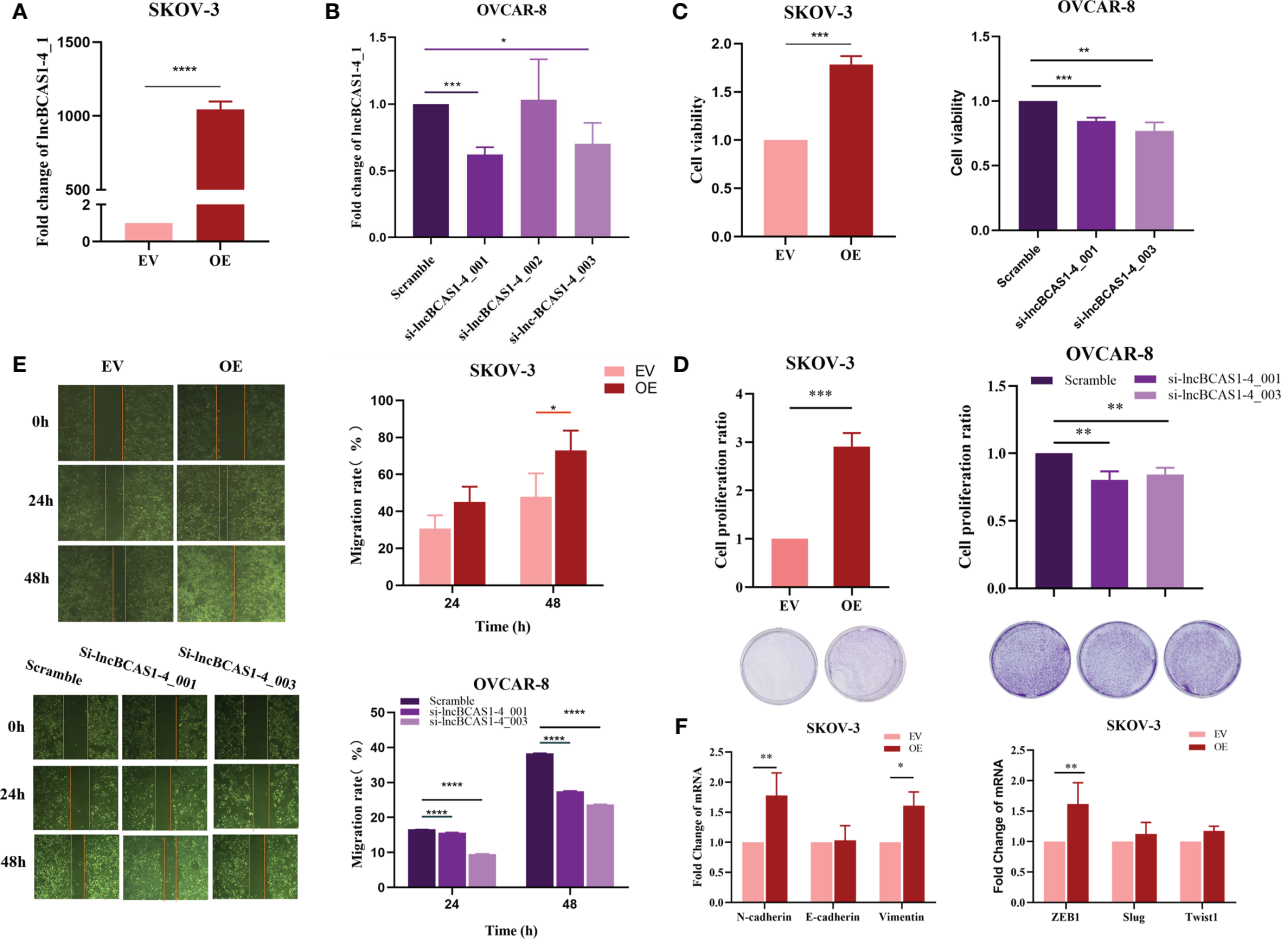
Effect of lncBCAS1-4_1 on proliferation and migration in ovarian cancer cells. **(A)** Compared with empty vector control cells (EV), the expression of lncBCAS1-4_1 was upregulated in SKOV3 cells transfected by adenovirus (OE) for 72 h. **(B)** The level of lncBCAS1-4_1 was downregulated in OVCAR8 cells transfected by siRNA (sh-lncBCAS1-4_001 and sh-lncBCAS1-4_003) for 72 h, compared to scramble control. **(C)** The cell viability was detected by CCK-8 after cells were transfected for 72 h. **(D)** The proliferation activity was detected by colony formation assay after the cells were transfected for 72 h. **(E)** The migration capacity was measured by wound healing assay after the cells were transfected for 72 h. **(F)** The mRNA levels of EMT-related genes were determined using RT-PCR. *p < 0.05, **p < 0.01, ***p < 0.001, ****p < 0.0001.

To validate the function of lncBCAS-1_4-1, SKOV3 cells were used to build up lncBCAS1-4_1 gain-of-function cell models (**Figure 4A**), while OVCAR8 cells were used to build up lncBCAS1-4_1 loss-of-function cell models (**Figure 4B**). The results of CCK8 (**Figure 4C**) and platting efficiency (**Figure 4D**) assay showed that overexpressed lncBCAS1-4_1 promoted proliferation, while knockdown of lncBCAS1-4_1 inhibited proliferation. Similarly, we found that the gain of lncBCAS1-4_1 increased migration, and the loss of lncBCAS1-4_1 decreased cell migration (**Figure 4E**). We then detected the expression of mRNAs associated with the EMT signaling pathway. The result demonstrated that overexpression of lncBCAS1-4_1 significantly upregulated the EMT mesenchymal marker including N-cadherin and Vimentin, as well as the EMT-related transcriptional factor (ZEB1) (**Figure 4F**).

### The Inhibition of 1α,25(OH)_2_D_3_ on Proliferation and Migration of Ovarian Cancer Cells Is Disrupted by lncBCAS-1_4-1

**Figure 5:**
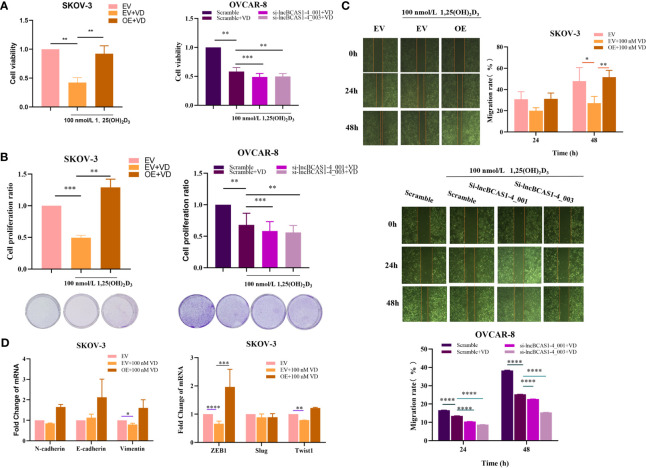
Effect of lncBCAS1-4_1 on proliferation and migration in ovarian cancer cells treated with 1α,25(OH)_2_D_3_. **(A)** The cell viability was detected by CCK-8 after lncBCAs1-4-gain SKOV3 and -loss OVCAR8 cells treated with 1α,25(OH)_2_D_3_ for 48 h. **(B)** The proliferation activity was detected by colony formation assay, after lncBCAs1-4-gain SKOV3 and -loss OVCAR8 cells treated with 1α,25(OH)_2_D_3_ for 48 h. **(C)** The migration capacity was measured by wound healing assay, after lncBCAs1-4-gain SKOV3 and -loss OVCAR8 cells treated with 1α,25(OH)_2_D_3_ for 48 h. **(D)** The mRNA levels of EMT-related genes were measured using RT-PCR. *p < 0.05, **p < 0.01, ***p < 0.001, ****p < 0.0001.

To ascertain the impact of lncBCAS1-4_1 on the antitumor action of vitamin D, ovarian cancer cells treated with 1α,25(OH)_2_D_3_ were interfered or overexpressed by the siRNA or adenovirus vector of lncBCAS1-4_1, respectively. As expected, the knockdown of lncBCAS1-4_1 significantly enhanced the 1α,25(OH)_2_D_3_ mediated antitumor effect, while overexpressed lncBCAS1-4_1 resisted the antitumor effect of 1α,25(OH)_2_D_3_ *in vitro* (**Figures 5A–C**). The results from **Figure 5D** showed that the expressions of Vimentin, ZEB1, and Twist1 were significantly reduced by 1α,25(OH)_2_D_3_ as compared to mock-vehicle negative control. However, the reduced ZEB1 levels in overexpressed lncBCAS1-4_1 SKOV3 cells were increased after treatment with 1α,25(OH)_2_D_3_. Taken together, these data indicated that the overexpression of lncBCAS1-4_1 significantly resisted the antitumor effect of 1α,25(OH)_2_D_3_, which was associated with upregulating ZEB1.

## Discussion

Previous reports demonstrated that 1α,25(OH)_2_D_3_ could regulate some protein-coding genes (31–33). Three studies showed that VDR regulated lncRNA profiling in skin and breast cancer (24–26). However, to date, there was no study investigating whether 1α,25(OH)_2_D_3_ could affect the expression pattern of lncRNAs in ovarian cancer. In the present study, 606 differentially expressed lncRNAs were dysregulated in 1α,25(OH)_2_D_3_-treated SKOV3 cells, in which 381 lncRNAs were upregulated and 225 lncRNAs were downregulated.

For DE-lncRNAs, we predicted their functions by clustering correlated mRNA and corresponding signaling pathways. Several DE-lncRNAs are closely associated with tumor development and progression, notably the Ras, MAPK, TGF-β, Rap1, PI3K-Akt, regulating pluripotency stem cells, and Hippo signaling pathways. It has been widely reported that 1α,25(OH)_2_D_3_ could inhibit TGF-β-induced EMT (19–26, 31–46) due to the fact that 1α,25(OH)_2_D_3_ inhibits β-catenin transcriptional activity by promoting VDR binding to β-catenin and inducing E-cadherin expression. Zerr et al. demonstrated that VDR was a negative regulator of the TGF-β/SMAD signaling pathway (47). 1α,25(OH)_2_D_3_ has inhibitory effect on cancer development through regulating MAPK and PI3K−Akt signaling pathways (21–23). In addition, there are also many lncRNAs that are predicted to be associated with the classic function of vitamin D by regulating glutathione metabolism, osteoclast differentiation, and cholesterol metabolism in this study. In a word, 1α,25(OH)_2_D_3_ dysregulated lncRNAs are likely to participate in some predicted tumor associated progression or some classical function of vitamin D. These associations are supposed to provide new orientations to explore the role of lncRNAs in tumor therapy and to improve vitamin D deficiency-related diseases.

The co-expression/regulatory networks of lncRNAs-mRNAs indicated that “TGF-β signaling pathway”, “epithelial cell proliferation”, and “Hippo signaling pathway” were significantly involved in 1α,25(OH)_2_D_3_ treated cancer cells. Up to date, there are lots of reports about how coding genes or non-coding genes to regulate EMT progress or EMT associated genes and transcription factors (48–51). Moreover, 1α,25(OH)_2_D_3_ was reported to have the effect on inhibiting the progression of EMT (31). Our results also showed that EMT signaling pathway was significantly activated in 1α,25(OH)_2_D_3_ treated ovarian cancer cells. It is plausible that these lncRNAs could mediate the EMT process by vitamin D signaling pathway, which supports our hypothesis that 1α,25(OH)_2_D_3_ has inhibitory effects on ovarian cancer cells by regulating lncRNA expression patterns.

In the present study, the most upregulated lncRNA was lncBCAS1-4_1, which has the closest relationship with the mRNA transcript of CYP24A1, because their 75% transcripts are the same. CYP24A1 is the gene coding the metabolic enzyme of 1α,25(OH)_2_D_3_, resulting in the loss of physiological activity by 1α,25(OH)_2_D_3_ (34). *In vitro* and *in vivo* studies also showed that CYP24A1 has been deemed as a candidate oncogene in many cancers, such as ovarian cancer (35), colorectal cancer (36, 37), prostate cancer (38), lung cancer (39), breast cancer (40), thyroid cancer (41), and so on. Moreover, a recent study showed that the upregulation of CYP24A1 and PFDN4 as well as nearby lncRNAs may be used as the potential diagnostic biomarker in colorectal cancer (52). Interestingly, it has been reported that mice with CYP24A1 knockout exhibited a fourfold reduction in thyroid tumor growth compared with wild-type CYP24A1 mice. They found that this phenotype was associated with the repression of the MAPK, PI3K/Akt, and TGFβ signaling pathways, and a loss of EMT in CYP24A1 knockout cells was also associated with the downregulation of genes involved in EMT, tumor invasion, and metastasis (53). Furthermore, functional analysis revealed that the TGF-β pathway was associated with lncBCAS1-4_1. Based on the 75% similarity with CYP24A1 and the relationship between CYP24A1 and EMT, as well as the key role of TGF-β in the EMT process, we focused on the link of lncBCAS1-4_1 and EMT. For the lncBCAS1-4_1 loss/gain cell model, the oncogenic role of lncBCAS1-4_1 was validated *in vitro*, and the overexpression of lncBCAS1-4_1 significantly resisted the antitumor effect of 1α,25(OH)_2_D_3_, which was associated with upregulated ZEB1. Thus, it was worthy to reveal the molecular mechanism of EMT-related lncRNAs in cancer and to demonstrate that lncBCAS1-4_1 can be a potential therapeutic target for patients.

Additionally, we also found that the most downregulated lncRNA (lnc-RWDD4-5_1) and IGFBP3 mRNAs were negatively correlated. After treatment with 1α,25(OH)_2_D_3_, the expression of lnc-RWDD4-5_1 was dramatically decreased, while that of IGFBP3 was increased (2.2-fold change). The most hub gene in the PPI network was IGF1, which can bind to IGFBP3. IGF1 and its binding proteins can promote cellular proliferation and inhibit apoptosis. *In vitro* studies showed that IGF1 increased ovarian cell growth and invasive potential (42). It is well documented that high IGF1 levels are significantly associated with early-stage cancer, nonserous histology, and optimal cytoreduction in epithelial ovarian cancers (43–45). Considerably, it is noteworthy that the most downregulated lncRNA (lnc-RWDD4-5_1) has a potential relationship with the hub gene (IGF1). However, the potential molecular mechanisms needed to be further verified.

There are also a couple of limitations to this study. Firstly, although the SKOV3 cell line is a useful model of ovarian cancer cells, it could not be used to predict the performance of 1α,25(OH)_2_D_3_ in actual tumors. And the action of 1α,25(OH)_2_D_3_ refers to different sets of genes in different cell lines (54–56). Secondly, the expressions of lncRNAs and mRNAs were analyzed by ovarian cancer cells, and further testing of these in the tumor tissues of patients is needed. Thirdly, the relationships among noncoding RNAs, mRNAs, and proteins need to be further investigated using bioinformatic prediction to understand their full function. Nevertheless, this is the first study of lncRNA expression patterns regulated by 1α,25(OH)_2_D_3_ in an ovarian cancer cell model, providing important basic data to support future work.

## Conclusions

In summary, we identified the 606 DE lncRNAs and 102 DE mRNAs in 1α,25(OH)_2_D_3_-treated ovarian cancer cells, which were mainly enriched in the cancer-related and vitamin D-related pathway. Moreover, by the lncBCAS1-4_1-mRNA core network, the EMT signal was identified, indicating the linkage of lncBCAS1-4_1 between EMT and vitamin D signaling. Furthermore, we established the lncBCAS1-4_1 loss/gain cell model and found that lncBCAS1-4_1 could abolish the antitumor effect of 1α,25(OH)_2_D_3_, which was associated with upregulating ZEB1. These data provide new evidence that lncRNAs can serve as targets for the antitumor effect of 1α,25(OH)_2_D_3_.

## Data Availability Statement

The datasets presented in this study can be found in online repositories. The names of the repository/repositories and accession number(s) can be found below: GEO Database and accession number GSE17363 https://www.ncbi.nlm.nih.gov/geo/query/acc.cgi?acc=GSE173633.

## Author Contributions

Conceptualization, HP and BL. Methodology, YX. Software, PW. Validation, YX, FJ, and JY. Formal analysis, PW and YX. Investigation, YX. Data curation, YX and JY. Writing—original draft preparation, YX, JY, HD, and FJ. Writing—review and editing, ZZ, HP, and BL. supervision, BYL. Project administration, ZZ, HP, and BL. Funding acquisition, FJ and BL. All authors contributed to the article and approved the submitted version.

## Funding

This research was supported by the National Natural Science Foundation of China (81872622 and 81703209) and a subproject of the National Key Research and Development Project (2018YFC0115703), and a project funded by the Priority Academic Program Development of Jiangsu Higher Education Institutions (PAPD).

## Conflict of Interest

The authors declare that the research was conducted in the absence of any commercial or financial relationships that could be construed as a potential conflict of interest.

## Publisher’s Note

All claims expressed in this article are solely those of the authors and do not necessarily represent those of their affiliated organizations, or those of the publisher, the editors and the reviewers. Any product that may be evaluated in this article, or claim that may be made by its manufacturer, is not guaranteed or endorsed by the publisher.

## References

[1] Sung PL Chang YH Chao KC Chuang CM. Global Distribution Pattern of Histological Subtypes of Epithelial Ovarian Cancer: A Database Analysis and Systematic Review. Gynecol Oncol (2014) 133(2):147–54. 10.1016/j.ygyno.2014.02.016 24556058

[2] Allemani C Weir HK Carreira H Harewood R Spika D Wang XS. Global Surveillance of Cancer Survival 1995-2009: Analysis of Individual Data for 25,676,887 Patients From 279 Population-Based Registries in 67 Countries (CONCORD-2). Lancet (9972) 2015:977–1010:385. 10.1016/S0140-6736(14)62038-9 PMC4588097 25467588

[3] Bhan A Soleimani M Mandal SS. Long Noncoding RNA and Cancer: A New Paradigm. Cancer Res (2017) 77(15):3965–81. 10.1158/0008-5472.CAN-16-2634 PMC8330958 28701486

[4] Yao RW Wang Y Chen LL. Cellular Functions of Long Noncoding RNAs. Nat Cell Biol (2019) 21(5):542–51. 10.1038/s41556-019-0311-8 31048766

[5] Lin C Yang L. Long Noncoding RNA in Cancer: Wiring Signaling Circuitry. Trends Cell Biol (2018) 28(4):287–301. 10.1016/j.tcb.2017.11.008 29274663 PMC5869122

[6] Xu J Wu J Fu C Teng F Liu S Dai C. Multidrug Resistant lncRNA Profile in Chemotherapeutic Sensitive and Resistant Ovarian Cancer Cells. J Cell Physiol (2018) 233(6):5034–43. 10.1002/jcp.26369 29219179

[7] Liu X Dai C Jia G Xu S Fu Z Xu J. Microarray Analysis Reveals Differentially Expressed lncRNAs in Benign Epithelial Ovarian Cysts and Normal Ovaries. Oncol Rep (2017) 38(2):799–808. 10.3892/or.2017.5741 28656240 PMC5562051

[8] Cui D Ma J Liu Y Lin K Jiang X Qu Y. Analysis of Long non-Coding RNA Expression Profiles Using RNA Sequencing in Ovarian Endometriosis. Gene (2018) 673:140–8. 10.1016/j.gene.2018.06.046 29920364

[9] Lu YM Wang Y Liu SQ Zhou MY Guo YR. Profile and Validation of Dysregulated Long Noncoding RNAs and mRNAs in Ovarian Cancer. Oncol Rep (2018) 40(5):2964–76. 10.3892/or.2018.6654 30132558

[10] Wang H Fu Z Dai C Cao J Liu X Xu J. LncRNAs Expression Profiling in Normal Ovary, Benign Ovarian Cyst and Malignant Epithelial Ovarian Cancer. Sci Rep (2016) 6:38983. 10.1038/srep38983 27941916 PMC5150236

[11] Hosseini ES Meryet-Figuiere M Sabzalipoor H Kashani HH Nikzad H Asemi Z. Dysregulated Expression of Long Noncoding RNAs in Gynecologic Cancers. Mol Cancer (2017) 16(1):107. 10.1186/s12943-017-0671-2 28637507 PMC5480155

[12] Zhou M Sun Y Sun Y Xu W Zhang Z Zhao H. Comprehensive Analysis of lncRNA Expression Profiles Reveals a Novel lncRNA Signature to Discriminate Nonequivalent Outcomes in Patients With Ovarian Cancer. Oncotarget (2016) 7(22):32433–48. 10.18632/oncotarget.8653 PMC5078024 27074572

[13] Guo L Peng Y Meng Y Liu Y Yang S Jin H. Expression Profiles Analysis Reveals an Integrated miRNA-lncRNA Signature to Predict Survival in Ovarian Cancer Patients With Wild-Type BRCA1/2. Oncotarget (2017) 8(40):68483–92. 10.18632/oncotarget.19590 PMC5620272 28978132

[14] Ding Y Yang DZ Zhai YN Xue K Xu F Gu XY. Microarray Expression Profiling of Long non-Coding RNAs in Epithelial Ovarian Cancer. Oncol Lett (2017) 14(2):2523–30. 10.3892/ol.2017.6448 PMC5530123 28781691

[15] Liu R Zeng Y Zhou CF Wang Y Li X Liu ZQ. Long Noncoding RNA Expression Signature to Predict Platinum-Based Chemotherapeutic Sensitivity of Ovarian Cancer Patients. Sci Rep (2017) 7(1):18. 10.1038/s41598-017-00050-w 28154416 PMC5428368

[16] Gao C Zhao D Zhao Q Dong D Mu L Zhao X. Microarray Profiling and Co-Expression Network Analysis of lncRNAs and mRNAs in Ovarian Cancer. Cell Death Discovery (2019) 5:93. 10.1038/s41420-019-0173-7 31098301 PMC6504870

[17] Shen L Liu W Cui J Li J Li C. Analysis of Long Non-Coding RNA Expression Profiles in Ovarian Cancer. Oncol Lett (2017) 14(2):1526–30. 10.3892/ol.2017.6283 PMC5529754 28789375

[18] Lou Y Jiang H Cui Z Wang X Wang L Han Y. Gene Microarray Analysis of lncRNA and mRNA Expression Profiles in Patients With Highgrade Ovarian Serous Cancer. Int J Mol Med (2018) 42(1):91–104. 10.3892/ijmm.2018.3588 29577163 PMC5979786

[19] Deeb KK Trump DL Johnson CS. Vitamin D Signalling Pathways in Cancer: Potential for Anticancer Therapeutics. Nat Rev Cancer (2007) 7(9):684–700. 10.1038/nrc2196 17721433

[20] Feldman D Krishnan AV Swami S Giovannucci E Feldman BJ. The Role of Vitamin D in Reducing Cancer Risk and Progression. Nat Rev Cancer (2014) 14(5):342–57. 10.1038/nrc3691 24705652

[21] Hmama Z Nandan D Sly L Knutson KL Herrera-Velit P Reiner NE. 1alpha,25-Dihydroxyvitamin D(3)-Induced Myeloid Cell Differentiation Is Regulated by a Vitamin D Receptor-Phosphatidylinositol 3-Kinase Signaling Complex. J Exp Med (1999) 190(11):1583–94. 10.1084/jem.190.11.1583 PMC2195730 10587349

[22] Meeker S Seamons A Paik J Treuting PM Brabb T Grady WM. Increased Dietary Vitamin D Suppresses MAPK Signaling, Colitis, and Colon Cancer. Cancer Res (2014) 74(16):4398–408. 10.1158/0008-5472.CAN-13-2820 PMC4134774 24938764

[23] Zhang X Zanello LP. Vitamin D Receptor-Dependent 1 Alpha,25(OH)2 Vitamin D3-Induced Anti-Apoptotic PI3K/AKT Signaling in Osteoblasts. J Bone Miner Res (2008) 23(8):1238–48. 10.1359/jbmr.080326 PMC2680173 18410228

[24] Jiang YJ Bikle DD. LncRNA Profiling Reveals New Mechanism for VDR Protection Against Skin Cancer Formation. J Steroid Biochem Mol Biol (2014) 144 Pt A:87–90. 10.1016/j.jsbmb.2013.11.018 24342142

[25] Kholghi Oskooei V Ghafouri-Fard S Omrani Mir D. A Combined Bioinformatics and Literature Based Approach for Identification of Long Non-Coding RNAs That Modulate Vitamin D Receptor Signaling in Breast Cancer. Klin Onkol (2018) 31(4):264–9. 10.14735/amko2018264 30541308

[26] Kholghi Oskooei V Geranpayeh L Omrani MD Ghafouri-Fard S. Assessment of Functional Variants and Expression of Long Noncoding RNAs in Vitamin D Receptor Signaling in Breast Cancer. Cancer Manag Res (2018) 10:3451–62. 10.2147/CMAR.S174244 PMC6140719 30254488

[27] Kuleshov MV Jones MR Rouillard AD Fernandez NF Duan Q Wang Z. Enrichr: A Comprehensive Gene Set Enrichment Analysis Web Server 2016 Update. Nucleic Acids Res (2016) 44(W1):W90–7. 10.1093/nar/gkw377 PMC4987924 27141961

[28] Ciulei G Orasan OH Coste SC Cozma A Negrean V Procopciuc LM. Vitamin D and the Insulin-Like Growth Factor System: Implications for Colorectal Neoplasia. Eur J Clin Invest (2020) 50(9):e13265. 10.1111/eci.13265 32379895

[29] Shirvani-Farsani Z Behmanesh M Mohammadi SM Naser A. Vitamin D Levels in Multiple Sclerosis Patients: Association With TGF-Beta2, TGF-betaRI, and TGF-betaRII Expression. Life Sci (2015) 134:63–7. 10.1016/j.lfs.2015.05.017 26037400

[30] Gisbert-Ferrandiz L Cosin-Roger J Hernandez C Macias-Ceja DC Ortiz-Masia D Salvador P. The Vitamin D Receptor Taq I Polymorphism Is Associated With Reduced VDR and Increased PDIA3 Protein Levels in Human Intestinal Fibroblasts. J Steroid Biochem Mol Biol (2020) 202:105720. 10.1016/j.jsbmb.2020.105720 32565249

[31] Hou YF Gao SH Wang P Zhang HM Liu LZ Ye MX. 1alpha,25(OH)(2)D(3) Suppresses the Migration of Ovarian Cancer SKOV-3 Cells Through the Inhibition of Epithelial-Mesenchymal Transition. Int J Mol Sci (2016) 17(8). 10.3390/ijms17081285 PMC5000682 27548154

[32] Huang J Yang G Huang Y Zhang S. Inhibitory Effects of 1,25(OH)2D3 on the Proliferation of Hepatocellular Carcinoma Cells Through the Downregulation of HDAC2. Oncol Rep (2017) 38(3):1845–50. 10.3892/or.2017.5848 28737824

[33] Schwartz Z Pedrozo HA Sylvia VL Gomez R Dean DD Boyan BD. 1alpha,25-(OH)2D3 Regulates 25-Hydroxyvitamin D3 24R-Hydroxylase Activity in Growth Zone Costochondral Growth Plate Chondrocytes *via* Protein Kinase C. Calcif Tissue Int (2001) 69(6):365–72. 10.1007/s00223-001-1009-y 11800234

[34] Luo W Johnson CS Trump DL. Vitamin D Signaling Modulators in Cancer Therapy. Vitam Horm (2016) 100:433–72. 10.1016/bs.vh.2015.11.004 26827962

[35] Kloss M Fischer D Thill M Friedrich M Cordes T Salehin D. Calcidiol and Calcitriol Regulate Vitamin D Metabolizing Enzymes in Cervical and Ovarian Cancer Cells. Anticancer Res (2010) 30(11):4429–34. 21115889

[36] Hobaus J Tennakoon S Heffeter P Groeschel C Aggarwal A Hummel DM. Impact of CYP24A1 Overexpression on Growth of Colorectal Tumour Xenografts in Mice Fed With Vitamin D and Soy. Int J Cancer (2016) 138(2):440–50. 10.1002/ijc.29717 PMC4832261 26238339

[37] Horvath HC Lakatos P Kosa JP Bacsi K Borka K Bises G. The Candidate Oncogene CYP24A1: A Potential Biomarker for Colorectal Tumorigenesis. J Histochem Cytochem (2010) 58(3):277–85. 10.1369/jhc.2009.954339 PMC2825493 19901270

[38] Luo W Yu WD Ma Y Chernov M Trump DL Johnson CS. Inhibition of Protein Kinase CK2 Reduces Cyp24a1 Expression and Enhances 1,25-Dihydroxyvitamin D(3) Antitumor Activity in Human Prostate Cancer Cells. Cancer Res (2013) 73(7):2289–97. 10.1158/0008-5472.CAN-12-4119 PMC3618587 23358686

[39] Shiratsuchi H Wang Z Chen G Ray P Lin J Zhang Z. Oncogenic Potential of CYP24A1 in Lung Adenocarcinoma. J Thorac Oncol (2017) 12(2):269–80. 10.1016/j.jtho.2016.10.010 27793774

[40] Osanai M Lee GH. CYP24A1-Induced Vitamin D Insufficiency Promotes Breast Cancer Growth. Oncol Rep (2016) 36(5):2755–62. 10.3892/or.2016.5072 27600601

[41] Hu N Zhang H. CYP24A1 Depletion Facilitates the Antitumor Effect of Vitamin D3 on Thyroid Cancer Cells. Exp Ther Med (2018) 16(4):2821–30. 10.3892/etm.2018.6536 PMC6143870 30233662

[42] Khandwala HM McCutcheon IE Flyvbjerg A Friend KE. The Effects of Insulin-Like Growth Factors on Tumorigenesis and Neoplastic Growth. Endocr Rev (2000) 21(3):215–44. 10.1210/edrv.21.3.0399 10857553

[43] Terry KL Tworoger SS Gates MA Cramer DW Hankinson SE. Common Genetic Variation in IGF1, IGFBP1 and IGFBP3 and Ovarian Cancer Risk. Carcinogenesis (2009) 30(12):2042–6. 10.1093/carcin/bgp257 PMC2792318 19858071

[44] Peeters PH Lukanova A Allen N Berrino F Key T Dossus L. Its Major Binding Protein (IGFBP-3) and Epithelial Ovarian Cancer Risk: The European Prospective Investigation Into Cancer and Nutrition (EPIC). Endocr Relat Cancer (2007) 14(1):81–90. 10.1677/erc.1.01264 17395977

[45] Huang YF Cheng WF Wu YP Cheng YM Hsu KF Chou CY. Circulating IGF System and Treatment Outcome in Epithelial Ovarian Cancer. Endocr Relat Cancer (2014) 21(2):217–29. 10.1530/ERC-13-0274 24273235

[46] Pendas-Franco N Aguilera O Pereira F Gonzalez-Sancho JM Munoz A. Vitamin D and Wnt/beta-Catenin Pathway in Colon Cancer: Role and Regulation of DICKKOPF Genes. Anticancer Res (2008) 28(5A):2613–23. 19035286

[47] Zerr P Vollath S Palumbo-Zerr K Tomcik M Huang J Distler A. Vitamin D Receptor Regulates TGF-Beta Signalling in Systemic Sclerosis. Ann Rheum Dis (2015) 74(3):e20. 10.1136/annrheumdis-2013-204378 24448349

[48] Liu Y Xue M Du S Feng W Zhang K Zhang L. Competitive Endogenous RNA is an Intrinsic Component of EMT Regulatory Circuits and Modulates EMT. Nat Commun (2019) 10(1):1637. 10.1038/s41467-019-13370-4 30967542 PMC6456586

[49] Liang Y Zhang CD Zhang C Dai DQ. DLX6-AS1/miR-204-5p/OCT1 Positive Feedback Loop Promotes Tumor Progression and Epithelial-Mesenchymal Transition in Gastric Cancer. Gastric Cancer (2020) 23(2):212–27. 10.1007/s10120-019-01002-1 31463827

[50] Freihen V Ronsch K Mastroianni J Frey P Rose K Boerries M. SNAIL1 Employs Beta-Catenin-LEF1 Complexes to Control Colorectal Cancer Cell Invasion and Proliferation. Int J Cancer (2020) 146(8):2229–42. 10.1002/ijc.32644 31463973

[51] Dhamija S Diederichs S. From Junk to Master Regulators of Invasion: lncRNA Functions in Migration, EMT and Metastasis. Int J Cancer (2016) 139(2):269–80. 10.1002/ijc.30039 26875870

[52] Sadeghi H Nazemalhosseini-Mojarad E Sahebi U Fazeli E Azizi-Tabesh G Yassaee VR. Novel Long Noncoding RNAs Upregulation may Have Synergistic Effects on the CYP24A1 and PFDN4 Biomarker Role in Human Colorectal Cancer. J Cell Physiol (2021) 236(3):2051–7. 10.1002/jcp.29992 32743796

[53] Zou M Baitei EY BinEssa HA Al-Mohanna FA Parhar RS St-Arnaud R. Cyp24a1 Attenuation Limits Progression of Braf(V600E) -Induced Papillary Thyroid Cancer Cells and Sensitizes Them to BRAF(V600E) Inhibitor Plx4720. Cancer Res (2017) 77(8):2161–72. 10.1158/0008-5472.CAN-16-2066 PMC6317881 28242615

[54] Lin R Nagai Y Sladek R Bastien Y Ho J Petrecca K. Expression Profiling in Squamous Carcinoma Cells Reveals Pleiotropic Effects of Vitamin D3 Analog EB1089 Signaling on Cell Proliferation, Differentiation, and Immune System Regulation. Mol Endocrinol (2002) 16(6):1243–56. 10.1210/mend.16.6.0874 12040012

[55] Krishnan AV Shinghal R Raghavachari N Brooks JD Peehl DM Feldman D. Analysis of Vitamin D-Regulated Gene Expression in LNCaP Human Prostate Cancer Cells Using cDNA Microarrays. Prostate (2004) 59(3):243–51. 10.1002/pros.20006 15042599

[56] Zhang X Li P Bao J Nicosia SV Wang H Enkemann SA. Suppression of Death Receptor-Mediated Apoptosis by 1,25-Dihydroxyvitamin D3 Revealed by Microarray Analysis. J Biol Chem (2005) 280(42):35458–68. 10.1074/jbc.M506648200 PMC3249976 16093247

