# Supplementary material for: A Newly Identified lncBCAS1-4_1 Associated With Vitamin D Signaling and EMT in Ovarian Cancer Cells
Source: Front Oncol. 2021 Aug 5;11:691500. doi: 10.3389/fonc.2021.691500 (PMC8377733; doi:10.3389/fonc.2021.691500)
Supplement: Supplementary file 1 [file DataSheet_1.pdf]

Supplementary Data

A newly identified lncBCAS1-4_1 associated with Vitamin D signaling and EMT in ovarian cancer cells

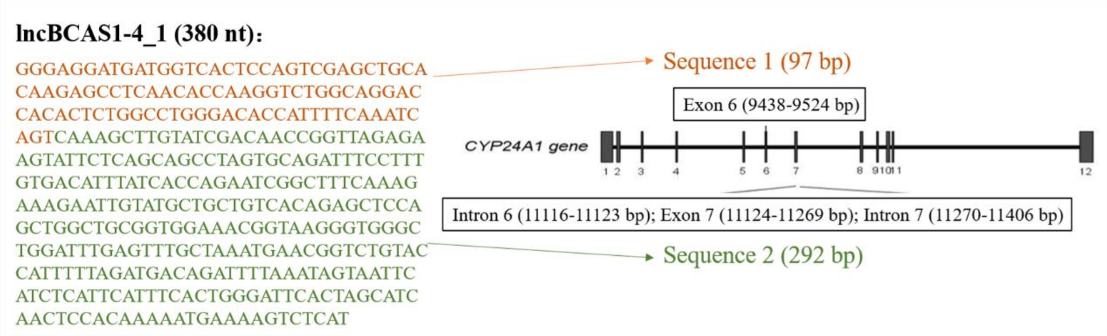

Figure S1. The most upregulated lncBCAS1-4_1 showed 75% same transcripts with CYP24A1. (http://genome.ucsc.edu/cgi-bin/hgTracks?db=hg38&lastVirtModeType=default&lastVirtModeExtraState=&virtModeType=default&virtMode=0&nonVirtPosition=&position=chr20%3A54161580%2D54169548&hgsid=1131787533_zJzaPkdZDmOvATlwbT3pHo04Ms15)

Table S1. Top 5 lncRNAs-mRNAs networks regulated by Vitamin D

top 5 lncRNAs	mRNA	correlation (r)
lnc-BCAS1-4:1	MAP7D2	0.9788
lnc-BCAS1-4:1	SLC44A5	0.9865
lnc-BCAS1-4:1	GEM	0.9798
lnc-BCAS1-4:1	TRPV6	0.9479
lnc-BCAS1-4:1	IGF1	-0.9654
lnc-BCAS1-4:1	APBB1IP	0.9653
lnc-BCAS1-4:1	COL1A1	0.9066
lnc-BCAS1-4:1	G6PD	0.9808
lnc-BCAS1-4:1	KLK6	0.9102
lnc-BCAS1-4:1	CCDC3	0.9027
lnc-BCAS1-4:1	KIT	0.9373
lnc-BCAS1-4:1	GRK5	0.9819
lnc-BCAS1-4:1	LOC100131756	0.9725
lnc-BCAS1-4:1	ATP8A1	0.9394
lnc-BCAS1-4:1	SLC16A2	0.9338
lnc-BCAS1-4:1	HS3ST1	0.9019
lnc-BCAS1-4:1	INHBB	0.9220
lnc-BCAS1-4:1	ID2	0.9583
lnc-BCAS1-4:1	PTPRR	0.9600
lnc-BCAS1-4:1	ID1	0.9350

lnc-BCAS1-4:1	CD97	0.9325
lnc-BCAS1-4:1	CXorf36	0.9293
lnc-BCAS1-4:1	ETS2	0.9758
lnc-BCAS1-4:1	IL18R1	0.9354
lnc-BCAS1-4:1	LXN	0.9178
lnc-BCAS1-4:1	TUBB3	0.9190
lnc-BCAS1-4:1	LSAMP	-0.9459
lnc-BCAS1-4:1	SRPX	0.9401
lnc-BCAS1-4:1	IGFBP3	0.9223
lnc-BCAS1-4:1	SIRPB1	0.9071
lnc-BCAS1-4:1	SIRPG	0.9139
lnc-BCAS1-4:1	CATSPER3	0.9897
lnc-BCAS1-4:1	FAM129A	0.9955
lnc-BCAS1-4:1	SCARA3	0.9453
lnc-BCAS1-4:1	NOV	-0.9230
lnc-BCAS1-4:1	BDNF	0.9169
lnc-BCAS1-4:1	CYP24A1	0.9913
lnc-CYP4F2-1:4	THBD	0.9231
lnc-CYP4F2-1:4	GEM	0.9034
lnc-CYP4F2-1:4	ID3	0.9507
lnc-CYP4F2-1:4	MIR205HG	-0.9525
lnc-CYP4F2-1:4	SLC37A2	0.9241
lnc-CYP4F2-1:4	TMPRSS4	-0.9096
lnc-CYP4F2-1:4	ID1	0.9239
lnc-CYP4F2-1:4	ITPK1	0.9251
lnc-CYP4F2-1:4	HES1	0.9591
lnc-CYP4F2-1:4	TUBB3	0.9106
lnc-CYP4F2-1:4	ANK1	-0.9495
lnc-CYP4F2-1:4	IGFBP1	0.9692
lnc-CYP4F2-1:4	IL20RB	-0.9607
lnc-CYP4F2-1:4	CDA	0.9163
lnc-CYP4F2-1:4	FAM46A	0.9400
lnc-CYP4F2-1:3	THBD	0.9440
lnc-CYP4F2-1:3	ID3	0.9844
lnc-CYP4F2-1:3	MIR205HG	-0.9774
lnc-CYP4F2-1:3	SLC37A2	0.9524
lnc-CYP4F2-1:3	GRK5	0.9012
lnc-CYP4F2-1:3	TMPRSS4	-0.9167
lnc-CYP4F2-1:3	SLC16A2	0.9193
lnc-CYP4F2-1:3	INHBB	0.9421
lnc-CYP4F2-1:3	GPCPD1	0.9292
lnc-CYP4F2-1:3	ID1	0.9427
lnc-CYP4F2-1:3	HES1	0.9380
lnc-CYP4F2-1:3	ANK1	-0.9831

lnc-CYP4F2-1:3	IGFBP1	0.9858
lnc-CYP4F2-1:3	SIRPB1	0.9044
lnc-CYP4F2-1:3	MSC	0.9298
lnc-CYP4F2-1:3	IL20RB	-0.9366
lnc-CYP4F2-1:3	CDA	0.9392
lnc-MSX2-4:9	GEM	0.9420
lnc-MSX2-4:9	TRPV6	0.9093
lnc-MSX2-4:9	GBP1	0.9109
lnc-MSX2-4:9	IGF1	-0.9630
lnc-MSX2-4:9	APBB1IP	0.9267
lnc-MSX2-4:9	COL1A1	0.9393
lnc-MSX2-4:9	G6PD	0.9566
lnc-MSX2-4:9	KLK6	0.9069
lnc-MSX2-4:9	GRK5	0.9452
lnc-MSX2-4:9	LOC100131756	0.9215
lnc-MSX2-4:9	ATP8A1	0.9045
lnc-MSX2-4:9	SLC16A2	0.9545
lnc-MSX2-4:9	ABCA1	-0.9360
lnc-MSX2-4:9	BCAS1	0.9759
lnc-MSX2-4:9	INHBB	0.9212
lnc-MSX2-4:9	ID2	0.9016
lnc-MSX2-4:9	PTPRR	0.9840
lnc-MSX2-4:9	ETS2	0.9931
lnc-MSX2-4:9	IL18R1	0.9023
lnc-MSX2-4:9	TUBB3	0.9566
lnc-MSX2-4:9	IGFBP1	0.9106
lnc-MSX2-4:9	LSAMP	-0.9023
lnc-MSX2-4:9	IGFBP3	0.9825
lnc-MSX2-4:9	SIRPB1	0.9574
lnc-MSX2-4:9	SIRPG	0.9585
lnc-MSX2-4:9	CATSPER3	0.9375
lnc-MSX2-4:9	IL20RB	-0.9090
lnc-MSX2-4:9	FAM129A	0.9718
lnc-MSX2-4:9	SCARA3	0.9080
lnc-MSX2-4:9	SIRPA	0.9496
lnc-MSX2-4:9	HHIP	-0.9261
lnc-MSX2-4:9	BDNF	0.9615
lnc-MSX2-4:9	CYP24A1	0.9606
lnc-MSX2-4:9	MAP7D2	0.9387
lnc-MSX2-4:9	SLC44A5	0.9361
lnc-MSX2-4:8	GEM	0.9513
lnc-MSX2-4:8	TRPV6	0.9126
lnc-MSX2-4:8	GBP1	0.9032
lnc-MSX2-4:8	IGF1	-0.9666

lnc-MSX2-4:8	APBB1IP	0.9262
lnc-MSX2-4:8	COL1A1	0.9349
lnc-MSX2-4:8	G6PD	0.9560
lnc-MSX2-4:8	KLK6	0.9012
lnc-MSX2-4:8	GRK5	0.9545
lnc-MSX2-4:8	LOC100131756	0.9263
lnc-MSX2-4:8	ATP8A1	0.9060
lnc-MSX2-4:8	SLC16A2	0.9596
lnc-MSX2-4:8	ABCA1	-0.9336
lnc-MSX2-4:8	BCAS1	0.9727
lnc-MSX2-4:8	INHBB	0.9283
lnc-MSX2-4:8	ID2	0.9138
lnc-MSX2-4:8	PTPRR	0.9846
lnc-MSX2-4:8	ID1	0.9057
lnc-MSX2-4:8	ETS2	0.9939
lnc-MSX2-4:8	IL18R1	0.9091
lnc-MSX2-4:8	TUBB3	0.9622
lnc-MSX2-4:8	IGFBP1	0.9201
lnc-MSX2-4:8	LSAMP	-0.9149
lnc-MSX2-4:8	IGFBP3	0.9790
lnc-MSX2-4:8	SIRPB1	0.9611
lnc-MSX2-4:8	SIRPG	0.9627
lnc-MSX2-4:8	CATSPER3	0.9438
lnc-MSX2-4:8	IL20RB	-0.9138
lnc-MSX2-4:8	FAM129A	0.9756
lnc-MSX2-4:8	SCARA3	0.9101
lnc-MSX2-4:8	SIRPA	0.9490
lnc-MSX2-4:8	HHIP	-0.9198
lnc-MSX2-4:8	BDNF	0.9543
lnc-MSX2-4:8	CYP24A1	0.9669
lnc-MSX2-4:8	MAP7D2	0.9425
lnc-MSX2-4:8	SLC44A5	0.9387

Table S2. The top lncBCAS1-4_1-mRNAs networks regulated by Vitamin D

lncRNA	mRNAs	correlation (r)
lnc-BCAS1-4:1	MAP7D2	0.9788
lnc-BCAS1-4:1	SLC44A5	0.9865
lnc-BCAS1-4:1	THBD	0.8869
lnc-BCAS1-4:1	PRAMEF2	0.8888
lnc-BCAS1-4:1	GEM	0.9798
lnc-BCAS1-4:1	TRPV6	0.9479
lnc-BCAS1-4:1	ID3	0.8811
lnc-BCAS1-4:1	GBP1	0.8391
lnc-BCAS1-4:1	IGF1	-0.9654

lnc-BCAS1-4:1	PTGER2	0.8686
lnc-BCAS1-4:1	APBB1IP	0.9653
lnc-BCAS1-4:1	COL1A1	0.9066
lnc-BCAS1-4:1	MIR205HG	-0.8532
lnc-BCAS1-4:1	PRAMEF1	0.8841
lnc-BCAS1-4:1	G6PD	0.9808
lnc-BCAS1-4:1	TGFB2	0.8359
lnc-BCAS1-4:1	KLK6	0.9102
lnc-BCAS1-4:1	CCDC3	0.9027
lnc-BCAS1-4:1	SLC37A2	0.8646
lnc-BCAS1-4:1	MEGF6	-0.8482
lnc-BCAS1-4:1	LAMA4	0.8819
lnc-BCAS1-4:1	KIT	0.9373
lnc-BCAS1-4:1	GRK5	0.9819
lnc-BCAS1-4:1	TMPRSS4	-0.8746
lnc-BCAS1-4:1	LOC100131756	0.9725
lnc-BCAS1-4:1	ATP8A1	0.9394
lnc-BCAS1-4:1	SLC16A2	0.9338
lnc-BCAS1-4:1	SERPINA3	0.8338
lnc-BCAS1-4:1	HS3ST1	0.9019
lnc-BCAS1-4:1	TSKU	0.8263
lnc-BCAS1-4:1	ABCA1	-0.8647
lnc-BCAS1-4:1	BCAS1	0.8937
lnc-BCAS1-4:1	INHBB	0.9220
lnc-BCAS1-4:1	GPCPD1	0.8393
lnc-BCAS1-4:1	KIAA1324L	0.8438
lnc-BCAS1-4:1	IDH2	0.8813
lnc-BCAS1-4:1	ID2	0.9583
lnc-BCAS1-4:1	PTPRR	0.9600
lnc-BCAS1-4:1	ID1	0.9350
lnc-BCAS1-4:1	CD97	0.9325
lnc-BCAS1-4:1	CXorf36	0.9293
lnc-BCAS1-4:1	ETS2	0.9758
lnc-BCAS1-4:1	CHRNA3	0.8455
lnc-BCAS1-4:1	NR1D1	-0.8215
lnc-BCAS1-4:1	SRGN	0.8500
lnc-BCAS1-4:1	COL16A1	0.8283
lnc-BCAS1-4:1	IL18R1	0.9354
lnc-BCAS1-4:1	LXN	0.9178
lnc-BCAS1-4:1	ITPK1	0.8401
lnc-BCAS1-4:1	HES1	0.8578
lnc-BCAS1-4:1	TUBB3	0.9190
lnc-BCAS1-4:1	C8orf31	-0.8609
lnc-BCAS1-4:1	ANK1	-0.8772

lnc-BCAS1-4:1	NPFFR2	-0.8276
lnc-BCAS1-4:1	PRSS33	0.8767
lnc-BCAS1-4:1	IGFBP1	0.8899
lnc-BCAS1-4:1	LSAMP	-0.9459
lnc-BCAS1-4:1	SRPX	0.9401
lnc-BCAS1-4:1	SGPP2	0.8413
lnc-BCAS1-4:1	RGS22	-0.8652
lnc-BCAS1-4:1	SLPI	0.8447
lnc-BCAS1-4:1	STK32A	0.8927
lnc-BCAS1-4:1	IGFBP3	0.9223
lnc-BCAS1-4:1	SIRPB1	0.9071
lnc-BCAS1-4:1	HRCT1	0.8563
lnc-BCAS1-4:1	G0S2	0.8528
lnc-BCAS1-4:1	SIRPG	0.9139
lnc-BCAS1-4:1	MSC	0.8174
lnc-BCAS1-4:1	SULT1C2	0.8604
lnc-BCAS1-4:1	FXYD3	-0.8365
lnc-BCAS1-4:1	CATSPER3	0.9897
lnc-BCAS1-4:1	IL20RB	-0.8360
lnc-BCAS1-4:1	FAM129A	0.9955
lnc-BCAS1-4:1	AIG1	0.8321
lnc-BCAS1-4:1	GULP1	-0.8998
lnc-BCAS1-4:1	TPPP	-0.8167
lnc-BCAS1-4:1	SCARA3	0.9453
lnc-BCAS1-4:1	SIRPA	0.8652
lnc-BCAS1-4:1	HHIP	-0.8443
lnc-BCAS1-4:1	CDA	0.8799
lnc-BCAS1-4:1	FAM46A	0.8952
lnc-BCAS1-4:1	NOV	-0.9230
lnc-BCAS1-4:1	BDNF	0.9169
